# Perceived Socio-moral Climate and the Applicability of Signature Character Strengths at Work: a Study among Hospital Physicians

**DOI:** 10.1007/s11482-018-9697-x

**Published:** 2019-01-12

**Authors:** Thomas Höge, Cornelia Strecker, Melanie Hausler, Alexandra Huber, Stefan Höfer

**Affiliations:** 1 Institute of Psychology, University of Innsbruck, Innrain 52, 6020 Innsbruck, Austria; 2 Department of Medical Psychology, Medical University of Innsbruck, Innsbruck, Austria

**Keywords:** Character strengths, Signature strengths, Applicability of character strengths, Socio-moral climate, Work engagement, Well-being, Hospital physicians

## Abstract

Previous research demonstrated that the applicability of signature character strengths at work is associated with employee well-being. However, there is a lack of research on possible antecedents of the applicability of signature character strengths in the occupational domain. In this study we examined whether the perceived socio-moral climate of medical departments has a positive impact on the applicability of hospital physicians’ signature character strengths and whether it relates to work engagement, hedonic subjective well-being (SWB) and eudaimonic psychological well-being (PWB). Based on cross-sectional data of *N* = 165 hospital physicians in Austria, we tested mediation models with perceived socio-moral climate as predictor, applicability of signature character strengths as mediator, and work engagement, SWB and PWB as outcomes. Additionally, we collected longitudinal data (time-lag T1-T2: 6 months) from a sub-sample (*n* = 69) for testing the relationship between the perceived socio-moral climate and the applicability of signature character strengths over time. The cross-sectional results showed indirect effects of the perceived socio-moral climate on work engagement and eudaimonic well-being via the applicability of signature character strengths at work. Results from a cross-lagged panel analysis suggested an impact of socio-moral climate at T1 on the applicability of signature character strengths 6 months later (T2), but also an even stronger reversed effect of the applicability of signature character strengths at T1 on perceived socio-moral climate at T2.

## Introduction

Based on the paradigm of positive psychology (Gable and Haidt 2005; Seligman and Csikszentmihalyi 2000), the role of individuals’ *character strengths* in the conduct of a happy, fulfilling, and flourishing life attracted increasing scholarly attention (Niemiec 2013; Park et al. 2004; Peterson and Seligman 2004). During the last decade, a substantial body of research has demonstrated that character strengths and their application in the occupational domain are related to positive individual and organizational outcomes (e.g., Forest et al. 2012; Gander et al. 2012; Harzer and Ruch 2012, 2013; Hausler et al. 2017b; Littman-Ovadia and Steger 2010; Littman-Ovadia et al. 2017).

However, the existing research in this field focused on the potential positive *consequences* of using character strengths at work, largely disregarding potential *antecedents:* Are there specific work conditions or context factors increasing opportunities for employees to apply their character strengths in the workplace? Answering this question is crucial for designing and developing organizations and workplaces from the perspective of *positive organizational scholarship* (Cameron and Spreitzer 2011).

This study is one of the first addressing this research gap. We propose that a specific form of organizational climate – the so called *socio-moral climate* (SMC, Pircher Verdorfer et al. 2015; Weber et al. 2008) – is a social context factor increasing the applicability of individual character strengths at work, and therefore contributing to the well-being of employees. In our study we tested this proposition exemplarily among hospital physicians. Hospital physicians are an occupational group characterized by heavy workloads, a high risk of job burnout (e.g., Mata et al. 2015; Prins et al. 2007; Shanafelt et al. 2003; Wurm et al. 2016), as well as special moral challenges and moral distress in everyday work life (Kälvemark et al. 2004).

Based on cross-sectional data we tested mediation models, in which the perceived SMC of the medical departments was indirectly related to physicians’ work engagement and well-being via an increased applicability of their individually most pronounced character strengths (*signature character strengths*) at work (ASCS-W).

In a smaller sub-sample we analyzed whether the perceived SMC predicted ASCS-W over time (6 months later), applying a cross-lagged procedure.

Hence, this study contributes to existing research in two areas: Firstly, it is one of the first studies shedding light on a potential condition-related antecedent of ASCS-W. Secondly, it points out a potential mechanism how SMC in the workplace can positively affect employees’ work engagement and well-being.

## Character Strengths and the Applicability of Signature Character Strengths at Work

The question how human beings can conduct a “good”, fulfilling and happy life is a core element of philosophical and religious approaches during history and across cultures. Many of these approaches, like the ancient Greek Aristotelian philosophy, the medieval Christian theology of Thomas of Aquino, Buddhism, Taoism, Confucianism, and Islam stress the importance of *virtues* and *good character* for a fulfilling life. Comparisons between these approaches reveal a surprising convergence of virtues forming the “good character” (Dahlsgaard et al. 2005). Against this background and combining it with a genuine psychological perspective, Peterson and Seligman (2004) proposed the Values-In-Action (VIA) character strengths classification system, comprising 24 character strengths organized under six core virtues (wisdom, courage, humanity, justice, temperance, and transcendence). In this conceptualization character strengths are defined as “positive traits, reflected in thoughts, feelings, and behaviors” (Park et al. 2004; p.603) enabling individuals and institutions to flourish and thrive (Peterson and Seligman 2004). Character strengths are ethically valued in their own right and build a dispositional basis for morally competent actions (Park and Peterson 2006).

Peterson and Seligman (2004) claimed that everybody possesses about three to seven character strengths which are typical of this individual. These character strengths are termed *signature character strengths*. They should usually be most frequently endorsed and their display should be accompanied by a sense of ownership and authenticity (‘this is the real me’), feelings of excitement, invigoration and inevitability. There should be an intrinsic motivation to apply these signature character strengths and a yearning to act in accordance with these signature character strengths. Moreover, applying one’s signature strengths should be characterized by experiences of discovery and learning (Peterson and Seligman 2004).

It was hypothesized that particularly the application of these personal signature character strengths in different life domains (e.g., work or private life) has positive effects on the well-being of individuals, because such actions are most concordant with the self, personal goals and values, and associated with experiences of meaning, passion, and calling (Harzer and Ruch 2012; Forest et al. 2012; Linley et al. 2010). Indeed, various empirical studies have demonstrated that the application of individual *signature character strengths* at work (ASCS-W) relates to job satisfaction, meaning at work, work engagement, calling, organizational citizenship behavior (OCB), less counterproductive work behavior (CWB), harmonious passion, life satisfaction, and psychological well-being (Forest et al. 2012; Hausler et al. 2017b; Huber et al. 2018 in this special issue; Harzer and Ruch 2012, 2013; Littman-Ovadia and Steger 2010; Littman-Ovadia et al. 2017).

In this study, we decided to focus solely on the applicability of *signature* character strengths at work and not on the applicability of *specific* character strengths and their function for hospital physicians´ work engagement and well-being. The main reason is the nature of the proposed antecedent (SMC): We argue, that the positive function of SMC is to give individuals room to act according to their own, *idiosyncratic* character formation and not to foster the application of *specific* character strengths at work.

## Socio-moral Climate in Organizations

As already stated above, we propose that a perceived SMC is a social context factor at work, which is able to increase the applicability of individual signature character strengths at work (ASCS-W) and therefore contributes to employee well-being. Before we describe the construct of SMC, we will clarify our usage of the terms “morale / moral” and “ethics / ethical” considering that a comprehensive discussion of definitions of these terms is far beyond the scope of this article. There is no consensus in the philosophical literature. Our usage follows the Aristotelian tradition meaning that “ethical” refers to the philosophical reflection about morale and its justification (i.e., a philosophical discipline), whereas “moral” refers to the specific sets of normative rules defining what is right and wrong (Höffe 2002). Thus, we use “moral” when individuals’ competences, orientations or behaviors are denoted, and we use “ethical” when general principles or philosophical reflections are denoted.

The concept of SMC has its roots in Kohlberg’s influential work on the development of moral competences in childhood and youth (Kohlberg 1984). In his ‘just-community-approach’ to moral development in schools, Kohlberg and colleagues stressed the role of a specific social school and classroom climate – the so called ‘moral atmosphere’ (Higgins 1995; Kohlberg 1985; Power et al. 1989). The concept was adapted to the occupational domain by Lempert (1994) and was further developed into the construct of SMC by Weber et al. 2008). SMC represents a sub-domain of the organizational climate referring to organizational practices of communication, teamwork, collective problem-solving and decision making, which follow rules of conduct based especially on Kantian ethics, Rawls’ theory of justice, and the discourse-ethics by Habermas (see Pircher Verdorfer et al. 2013a; Power et al. 1989). Such a climate should form a field of socialization for moral orientations and competences (Power et al. 1989; Weber et al. 2008).

A positive, beneficial SMC can be described by the following five components (Weber et al. 2008; Pircher Verdorfer et al. 2013a): (1) Open confrontation of the employees with conflict and constructive conflict resolution, (2) reliable and constant appreciation, care, and support by supervisors and colleagues, (3) open communication and participative cooperation, (4) trust-based assignment and allocation of responsibility corresponding to the respective employees’ capabilities, and (5) organizational concern for the individual. In short, SMC at work denotes a social climate in an organization, department or work team characterized by discursive, participative, appreciative, supportive, and caring interactions between supervisors and subordinates and among co-workers.

Empirical research on SMC established the following two antecedents of a high SMC: (1) participative organizational structures (Weber et al. 2009) and (2) a *servant* leadership style (Pircher Verdorfer et al. 2013b). A servant leadership style is characterized by the attitude that the most important leadership goal is not to increase the efficacy of the organization but to “serve”, to empower, and to develop the followers (Greenleaf 2002; Van Dierendonck 2011).

With respect to potential outcomes of a high SMC, studies demonstrated positive relations to prosocial, democratic and community related action orientations (Weber et al. 2009), organizational commitment, psychological ownership and low turnover intentions (Pircher Verdorfer et al. 2013a, b; Weber et al. 2009), lower organizational cynicism and deviance (Pircher Verdorfer et al. 2015), knowledge sharing (Pircher Verdorfer et al. 2013a), decision comprehensiveness and team innovation (Seyr and Vollmer 2014), the experience of meaning at work (Schnell et al. 2013), and work engagement (Pircher Verdorfer et al. 2013b). This study is the first linking SMC to the concepts of ASCS-W and aspects of general well-being.

## Research Hypotheses

Initial research on “moral atmosphere” in the school context (Kohlberg 1985) and SMC in occupational settings (Lempert 1994; Weber et al. 2008) was predominantly interested in potential socialization effects for developing moral competences, and prosocial, community related attitudes and behaviors. In this study we adopted a different perspective on the relation between SMC and virtuous behavior at work. We did not evaluate whether SMC at work contributes to the development of a “good character” or increases the level of some character strengths more than others, even though these are also promising research questions. Instead, we were interested in the relation between the level of SMC and the applicability of individuals’ existing idiosyncratic character strengths patterns (i.e., signature character strengths) in the work domain. The assumption that SMC will be positively related to ASCS-W can be derived from the following theoretical considerations:

Whether an individual applies her or his character strengths in a specific life domain or social setting depends on two conditions at least (Harzer and Ruch 2013): First, the individual needs to possess character strengths to a certain degree to show relevant behaviors. We call this the *dispositional prerequisite* of the application of character strengths. Second, the probability that individuals’ signature character strengths are displayed in overt behaviors in a specific situation should also be influenced by the degree to which situational characteristics allow or even reinforce and motivate such signature character strength driven behaviors. We call this the *condition-related prerequisite* of the application of character strengths. Based on the influential early contributions to the person-vs-situation debate by Mischel (1969, 1977), a broad scientific consensus has emerged that the *strength* or *weakness* of a situation influences the degree to which dispositions or traits are guiding behavior in this situation (Cooper and Withey 2009). A *strong situation* is characterized by situational pressures towards *specific* behaviors. It restricts individuals’ autonomy to choose between alternatives, which attenuates the behavior-guiding role of individual trait patterns (Mischel 1977).

Organizational work settings often create comparatively strong situations (Davis-Blake and Pfeffer 1989). The strongness depends, for example, on aspects like the flatness or depth of hierarchical structure, division of labor, narrowness of job descriptions and work roles, and the level of workers´ job control and possibilities to participate in organizational decision making (Davis-Blake and Pfeffer 1989). The more ‘restrictive’ the work system, the ‘stronger’ should be the situations constraining employees’ opportunities to display their individual personality and behavior styles and patterns (Meyer et al. 2010). Against this background, the concept of SMC encompasses climate aspects which are able to create comparatively weak situations at work. A high SMC is discursive and participative allowing or even encouraging employees –independently of their rank or formal position– to express their individual perspectives, opinions, and standpoints and to engage in organizational decisions. For example, in a medical department characterized by a discursive and participative work climate, each physician will be better able to make valuable contributions based on her or his individual signature strengths pattern. Whereas in a medical department characterized by a climate of command, obedience, and avoidance of open discussions the individual physician will be less able to make autonomous contributions fueled by her or his specific signature character strength pattern but behave in a more ‘standardized’ way according to the instructions and expectations expressed by others (e.g., supervisors). A fully developed SMC is also appreciative, supportive, and caring which means that there is a social climate accepting diversity and expressing respect and concern towards each individual. For example, in a medical department with a high SMC there should be a climate in which physicians appreciate individual differences in signature character strengths patterns and believe that all of these different patterns deserve respect and their display should be encouraged and supported. Thus, we infer that a high SMC will lead to better opportunities for hospital physicians to display their individual personality including their individual signature character strength pattern within their work role.

However, SMC does not only encompass aspects creating weak situations as described above. In addition, SMC should increase the probability that morally positive dispositions are developed and more applicable compared to immoral or antisocial behavior orientations (Weber et al. 2008). In this sense, SMC also includes aspects of a *strong* situation by establishing social practices and social norms in which the display of *specific* dispositions, namely virtuous and prosocial dispositions like *character strengths*, should be more accepted, more successful for the individual and more reinforced by interaction partners. Immoral and antisocial behaviors will be less successful and socially sanctioned. From this rationale, we derive the following *core hypothesis* of our study:

Hypothesis 1: The perceived socio-moral climate (SMC) of the medical department will be positively related to the applicability of hospital physicians’ signature character strengths at work (ASCS-W).

Based upon theoretical considerations and previous empirical findings, we hypothesize positive relations between ASCS-W, work engagement and well-being. The proposition that the application of signature strengths is positively related to engagement was already highlighted by Seligman (2002) in his *Authentic Happiness Theory* and in his more recent theory of *well-being* based upon the *PERMA-Model* (Seligman 2011). In both theories the experience of engagement in life is an important element of happiness and well-being. The *Authentic Happiness Theory* propounds three elements of happiness: positive emotions, engagement, and meaning, whereas the *PERMA-Model* as part of the more recent well-being theory includes five elements of well-being: positive emotions, engagement, positive relationships, meaning, and accomplishment. According to both theories the display of signature strengths in everyday life should effect the experience of an engaged life and therefore foster happiness and well-being respectively. These propositions should not only be valid for the display of signature strengths in general, but also when only the work domain is concerned (Harzer and Ruch 2013).

Indeed, the link between ASCS-W and work engagement was empirically demonstrated, for example, by Harzer and Ruch (2013) and Lavy and Littman-Ovadia (2017). A positive relationship with well-being was established, for example, by Linley et al. (2010), Forest et al. (2012) and in samples of medical students and resident physicians by Hausler et al. (2017b).

However, Seligman (2002, 2011) did not provide an elaborated theoretical explanation *why* the application of signature strengths should increase engagement and well-being. We argue that with respect to the work domain at least three theoretical approaches contribute to an understanding of the proposed relations between ACSC-W, work engagement and well-being: (1) the *Self-Determination-Theory* (Deci and Ryan 2000), (2) the concept of *self-concordance* (Sheldon and Kasser 1998), and (3) recent conceptualizations of *meaning* in work (Schnell et al. 2013).

Drawing upon the Self-Determination-Theory, we assume that the possibility of applying signature character strengths at work increases the satisfaction of the basic needs for autonomy, competence and with respect to social character strengths (e.g. love) also relatedness (Deci and Ryan 2000). This in turn affects work engagement and well-being positively (Linley et al. 2010). Physicians` experiences of applying their individual signature strengths at work should contribute to the satisfaction of their *need for autonomy*, because displaying one’s own signature strengths is intrinsically motivated (Peterson and Seligman 2004) and intrinsically motivated behavior is associated with the experience of autonomy (Deci and Ryan 2000). Applying one’s own signature strengths should also satisfy the *need for competence*, because applying a *strength* is by definition competent behavior (Peterson and Seligman 2004). Finally, the application of a specific subset of character strengths with social implications (e.g., love, kindness) at work should contribute to the satisfaction of the need for relatedness because it should lead to more social interactions of a higher quality and in the long run to stronger social bonds at the workplace.

Moreover, the ASCS-W reflects to what degree work goals and procedures are *self-concordant.* Acting in a self-concordant way affects motivation and well-being positively (Sheldon and Kasser 1998). This is also in line with current conceptualizations of *meaning* in work. Experiencing *coherence* between one’s own personality (incl. character strenghts) and aspects of the work situation constitutes –together with *direction*, *significance*, and *belonging*– the experience of *meaning* in work (Schnell et al. 2013). The experience of meaning in work is interwined with work engagement. Both can be understood as two sides of the same medal with meaning as the cognitive-evaluative side, and work engagement as the motivational-behavioral side (Höge and Schnell 2012). Hence, we propose the following Hypothesis 2:

Hypothesis 2: The applicability of signature character strengths at work (ASCS-W) will be positively related to hospital physicians’ work engagement.

With respect to the link between ASCS-W and well-being we distinguish between two forms of well-being: hedonic well-being and eudaimonic well-being (Ryan and Deci 2001). The psychological conceptualization of well-being follows two traditions: The *hedonic* tradition, focusing on the exploration of subjective well-being (SWB) or “happiness” (e.g., Diener 1984; Kahneman et al. 1999), defines well-being primarily as high levels of positive emotions, low levels of negative emotions, and a high degree of general satisfaction with one’s life (Deci and Ryan 2008). The *eudaimonic* tradition of research on well-being is associated with the term *psychological well-being* (PWB, e.g. Ryff 1989; Ryff and Keyes 1995). This tradition challenged the hedonic view on well-being with its focus on positive emotions and pleasure. Instead, scholars in the eudaimonic tradition, which reaches back to ideas of Aristotle, presented a multidimensional approach to well-being that covers distinct aspects of human self-actualization like autonomy, engagement, personal growth, self-acceptance, meaning, mastery, and positive relationships (Ryan and Deci 2000; Ryff and Keyes 1995). Although we assume that ASCS-W will be positively related to both dimensions of well-being, and both dimensions will be strongly interrelated, we propose that the link to eudaimonic PWB will be stronger than the one to hedonic SWB. Displaying virtuous behavior in terms of one’s own signature character strengths should very often lead to positive emotions and pleasure (SWB), but not necessarily. However, displaying one’s signature character strengths should necessarily lead to experiences of self-actualization, meaningfulness and mastery (Peterson and Seligman 2004) which are important components of PWB.

Indeed, Hausler et al. (2017c) reported stronger correlations between character strengths of medical students and PWB than SWB, and Hausler et al. (2017b) found a stronger relation between ASCS-W and PWB than SWB among medical students and resident physicians.

Hypothesis 3a: The applicability of signature character strengths at work (ASCS-W) will be positively related to hospital physicians’ eudaimonic psychological well-being (PWB).

Hypothesis 3b: The applicability of signature character strengths at work (ASCS-W) will be positively related to hospital physicians’ hedonic well-being (SWB).

Hypothesis 3c: The positive relationship between the applicability of signature character strengths at work (ASCS-W) and eudaimonic well-being (PWB) of hospital physicians will be stronger than the relationship with hedonic subjective well-being (SWB).

**Fig. 1:**
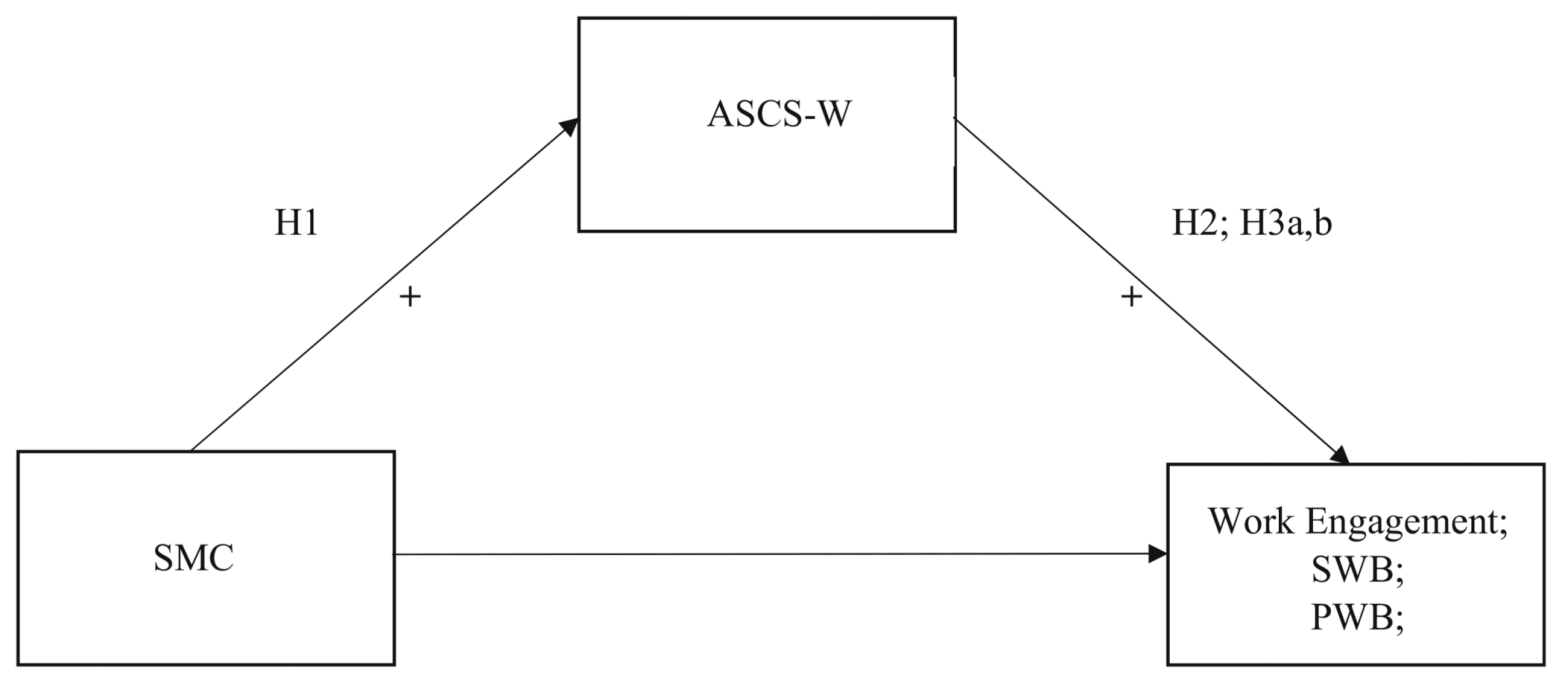
Hypothetical mediation model. *Note.* SMC: Socio-moral climate; ASCS-W: Applicability of signature character strengths at work; SWB: Subjective well-being (hedonic); PWB: Psychological well-being (eudaimonic)

From Hypotheses 1, 2, 3a, and 3b a mediational model can be derived, hypothesizing an indirect effect of perceived SMC on work engagement and both dimensions of well-being via ASCS-W (see Fig. 1).

Finally, we hypothesize that the relationship between SMC and ASCS-W (see Hypothesis 1) cannot only be established cross-sectionally but also longitudinally over a time period of 6 months. To test this hypothesis we will test a cross-lagged model (see Fig. 1). Such a cross-lagged analysis has several incremental benefits compared to the cross-sectional analysis based on Hypothesis 1. Although a cross-lagged analysis cannot demonstrate causality conclusively, the identification of a time-lagged relation of SMC at T1 and ASCS-W at T2 (controlled for ASCS-W at T1) increases the plausibility of the assumption that indeed SMC effects ASCS-W. Moreover, a cross-lagged design allows a simultaneous test of the reversed relation that ASCS-W at T1 predicts SMC at T2 controlled for SMC at T1. The identification of such a reversed relationship would definitely show that there must also be causal relations contributing to the cross-sectional correlation other than the assumed causal effect of SMC on ASCS-W. Finally, a longitudinal analysis with a time lag between the measurement of the proposed antecedent and outcome reduces the risk of inflated correlations caused by common method variance (Podsakoff et al. 2003).

Hypothesis 4: The Perceived socio-moral climate (SMC) of the medical department at T1 will positively predict the applicability of hospital physicians’ signature character strengths (ASCS-W) at T2 6 months later (controlled for ASCS-Wat T1).

## Method

### Sample and Procedure

The study was conducted in two hospitals of a regional hospital network including a large university hospital in Austria. Hospital physicians were invited via email to fill out an online questionnaire. Data collection was part of a larger research project on health and well-being of medical students and hospital physicians. After eliminating the data of 35 participants because of incomplete data in the variables of this study, the sample at T1 consisted of *N* = 165 physicians (response rate: 19.5%). About 64% of participants were female, about 36% were male. The mean age at T1 was 33.44 years (*SD* = 6.73, range = 24–64 years). A large majority of *n* = 145 were resident physicians in training, *n* = 20 were medical specialists. The physicians worked in medical departments concerned with 14 medical disciplines, e.g. internal medicine, trauma surgery, neurology, pediatrics, anesthesia, psychiatry, and radiology.

After a time period of 6 months all participants from T1 were invited to participate in a follow-up (T2). A total of *n* = 69 physicians also participated at T2. About 64% of these participants at T2 were female, about 36% were male. The mean age at T2 was 32.81 years (*SD* = 5.08, range = 25–51 years). Also at T2 a large majority of *n* = 63 were resident physicians in training, *n* = 6 were medical specialists. Participants at T1 and T2 filled out a questionnaire including the same measures.

### Measures

#### Applicability of Signature Character Strengths at Work (ASCS-W)

For measuring ASCS-W we used the two-step procedure developed and validated by Harzer and Ruch (2013). In the first step, participants’ levels of all 24 character strengths were measured with a German translation of the English 120-item short version of the *Values in Action Inventory of Strengths* (Höfer et al. 2018 in this special issue; Littman-Ovadia 2015; original: VIA Institute on Character 2014). The German items were taken from the German 240-item long version of the VIA-IS (Ruch et al. 2010). Psychometric properties were similar to the original 240 items version with a mean Cronbach’s alpha of .79 (range: .71 to .91; *SD* = 0.05). The five-point Likert-scale ranges from *strong disagreement* (=1) to *strong agreement* (=5).

In the second step, participants received feedback about their individual top five character strengths, which we assumed to be “proxies” for their individual *signature* character strengths. Peterson and Seligman (2004) proposed several additional criteria for signature character strengths beyond being top ranked. However, operationalizing signature character strengths only by being the top five (or top seven) strengths of an individual is a common procedure in the empirical literature on signature character strengths (e.g., Allan and Duffy 2014; Harzer and Ruch 2012, 2013; Linley et al. 2007, 2010).

Participants had to evaluate the applicability of their top five strengths at work using the German version of the *Applicability of Character Strengths Rating Scale (*ACS-RS) by Harzer and Ruch (2013). Participants responded to four items for each of their five signature character strengths (e.g., „I do it in my everyday work“). The response format was a five-point scale from *never* (=1) to (*almost) always* (=5). In this study internal consistency (Cronbach’s alpha) of the ACS-RS at work was .80 at T1, and .79 at T2.

#### Perceived Socio-moral Climate (SMC)

Participants’ perceptions of SMC were assessed using the German translation of the 21-item version of the *Socio-moral Climate Questionnaire* (SMCQ) by Pircher Verdorfer et al. (2015). The SMCQ measures five subscales of SMC: (1) open confrontation with conflicts, (2) respect, (3) open communication and participative cooperation, (4) allocation of responsibility, (5) organizational concern. Confirmatory factor analyses (CFAs) for the German version confirmed a second-order factor structure justifying the aggregation of the five sub-scales into a general SMC-score (Pircher Verdorfer et al. 2015). In this study, we focus only on the general SMC-score and not on sub-dimensions of SMC. The original items refer to the psychological climate of the whole organization. We adapted the items to reflect a medical department reference. Item examples for the five sub-dimensions are: (1) “In our medical department we deal openly with conflicts and disagreements”, (2) “Mutual respect is a central value in our medical department”, (3) “In our medical department you can speak your mind without fear of negative consequences” (4) “In our medical department everyone is tasked according to his/her skill set”, (5) “Our medical department attempts to meet the needs of all its members”. Responses were scored on a 5-point Likert scale ranging from strongly disagree (=1) to strongly agree (=5). Cronbach’s alpha of the composite score for SMC (mean across the subscale scores) was .93 at T1 and .91 at T2.

#### Work Engagement

Work Engagement was measured using the German nine-item short-version of the *Utrecht Work Engagement Scale* (UWES; Schaufeli and Bakker 2003; Schaufeli et al. 2006). Work engagement is defined as a positive, fulfilling work-related state of mind that is characterized by vigor, dedication, and absorption (Schaufeli et al. 2006). An item example is: “*At my job, I feel strong and vigorous*“. The response format was a seven-point scale ranging from never (=0) to always (=6). Cronbach’s alpha was .94 at T1 and .96 at T2 in this study.

#### Subjective and Psychological Well-Being

Hedonic SWB and eudaimonic PWB were measured using the German version of the *Comprehensive Inventory of Thriving* (CIT; Hausler et al. 2017a; original: Su et al. 2014). The CIT is the first well-being measure integrating the hedonic (SWB) and eudaimonic (PWB) approaches of conceptualizing and assessing well-being (Su et al. 2014). The CIT comprises 54 items measuring 18 sub-dimensions of SWB and PWB. CFAs based on the data of the German validation sample indicated a second-order factor structure with 18 first order factors and SWB and PWB as second-order factors (Hausler et al. 2017a). Three of the 18 sub-dimensions can be aggregated into a composite SWB-score: positive emotions, negative emotions (recoded), and life satisfaction. The other 15 sub-scales can be further summarized into six components: relationships, engagement, meaning in life, mastery, autonomy, and optimism, which in turn can be combined to one composite PWB-score (Hausler et al. 2017a). Item examples are: “I feel positive most of the time “(SWB; sub-dimension: positive feelings); “My life has a clear sense of purpose “(PWB, sub-dimension: meaning in life). Response format was a five-point Likert-scale ranging from *strongly disagree* (=1) to *strongly agree* (=5). In this study Cronbach’s alphas of the composite score of PWB were .92 (T1) and .94 (T2). Cronbach’s alphas of the composite score of SWB were .96 (T1) and .96 (T2).

### Statistical Analyses

Descriptives, intercorrelations and scale reliabilities (Cronbach’s alphas) were calculated with IBM SPSS Statistics Version 24. For estimating parameters of the mediation models (Hypotheses 1, 2, 3a, 3b; see Fig. 1) we used the SPSS-Macro PROCESS (Hayes 2013) applying multiple linear regressions and estimating indirect effects via bootstrapping (5000 bootstrap samples). For the comparison of the relations between ACSCS-W and SWB, and ASCS-W and PWB (Hypothesis 3c) we used a web-based calculator published by Lee and Preacher (2013). This calculator computes a correlations difference test based on Fisher’s Z transformation described in Steiger (1980).

The longitudinal Hypothesis 4, which stated an effect of SMC at T1 on ASCS-W at T2, was tested by computing a cross-lagged path-analysis with manifest variables (AMOS 24). This analysis was based on the sub-sample (*n* = 69) with available data from T1 and T2. Because the study design includes only two measurement points instead of three, longitudinal tests of the mediation models were not feasible.

## Results

Table 1 shows the means, standard deviations, and intercorrelations of all variables. None of our variables were significantly related to sex and age. First, we descriptively analyzed which five character strengths frequently were one of the top five signature character strengths of the participating physicians at T1: At 56% of physicians, *honesty* was a signature character strength, followed by *kindness* (48%), *love* (47%), *judgment* (46%), and *fairness* (36%). The five character strengths rarely occuring among individual signature strengths in our sample were: *self-regulation* (4%), *perspective* (4%), *spirituality* (5%), *prudence* (5%), and *leadership* (7%). Results in Table 1 also demonstrate that SMC and ASCS-W were not very stable over the six-month period: SMC at T1 correlated with SMC at T2 with *r* = .60 (*p* < .01) and ASCS-W at T1 correlated with ASCS-W at T2 with *r* = .55 (*p* < .01) indicating a shared variance of 36% and 30%, respectively.

**Fig. 2:**
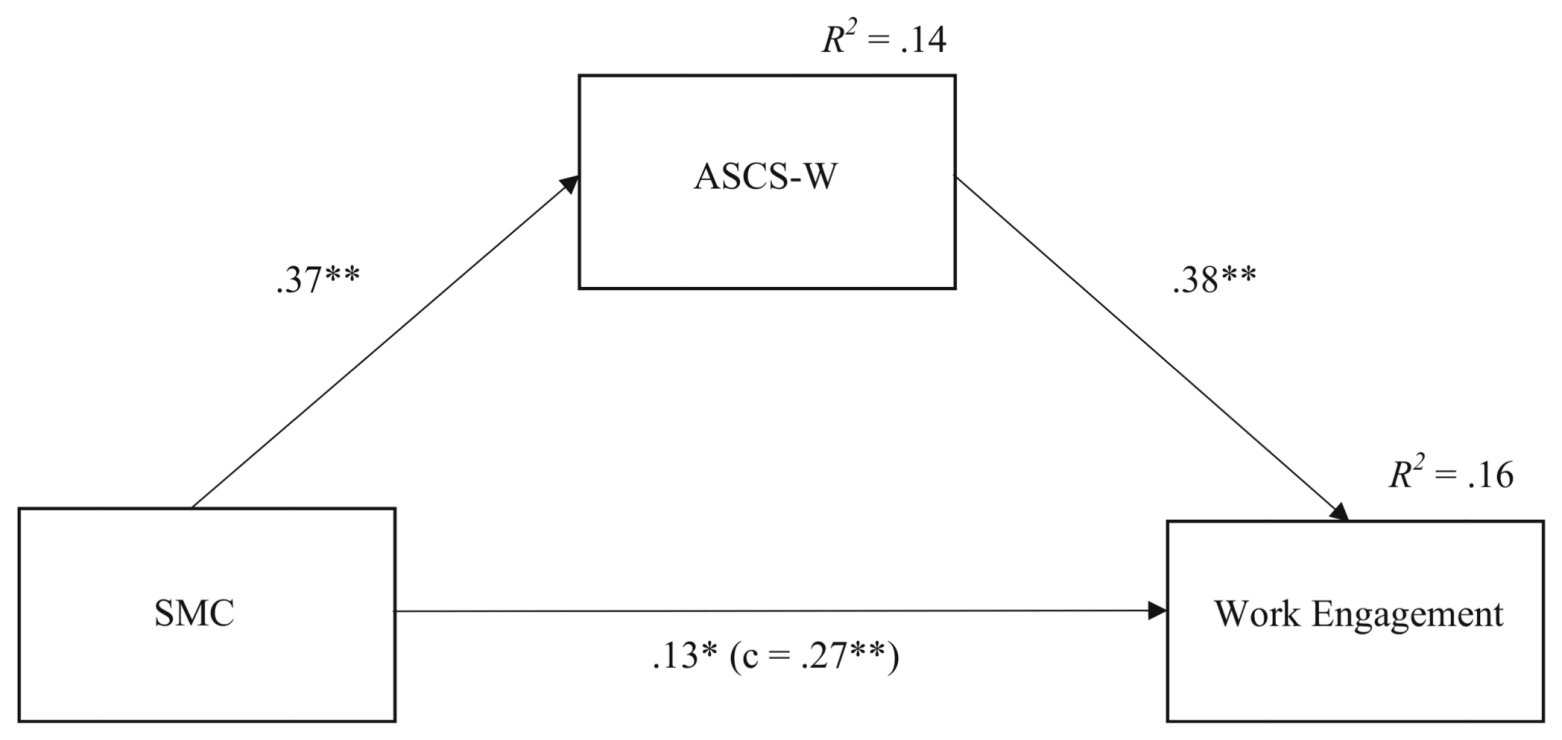
Results for the mediation model predicting work engagement (T1). *Note. N* = 165; *p < .05, ** *p* < .01, n.s.: non-significant (one-tailed); standardized regression coefficients; SMC: Socio-moral climate; ASCS-W: Applicability of signature character strengths at work *R^2^* = determination coefficient (explained variance); c: total effect without controlling for indirect effect

Table 2 and Fig. 2 present the results of the cross-sectional test (T1) of the mediation model with SMC as independent variable, ASCS-W as mediator, and work engagement as dependent variable (Hypotheses 1 and 2). The relationship between SMC and ASCS-W was significant, and ASCS-W was significantly related to work engagement. Results of a Sobel-Test and the bootstrap confidence interval not including zero indicate that the indirect effect of SMC on work engagement via ASCS-W is significant. Thus, Hypothesis 1 and Hypothesis 2 were confirmed.

**Fig. 3:**
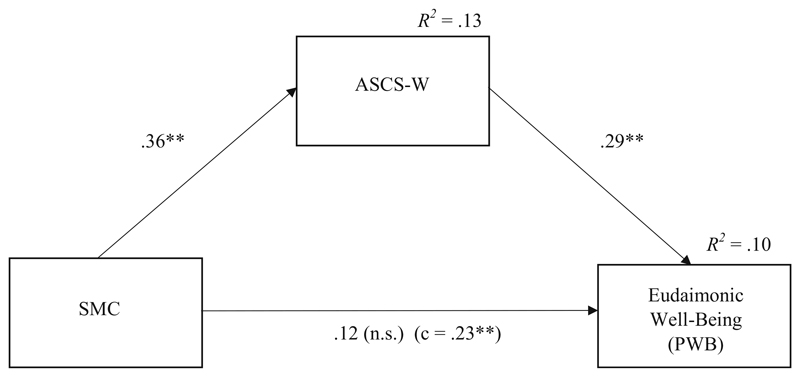
Results for the mediation model predicting PWB (T1). *Note. N* = 165; *p < .05, ** *p* < .01, n.s.: non-significant (one-tailed); standardized regression coefficients; SMC: Socio-moral climate; ASCS-W: Applicability of signature character strengths at work; *R^2^* = determination coefficient (explained variance)*;* c: total effect without controlling for indirect effect

Table 3 and Fig. 3 show the results of the cross-sectional mediation model with PWB as outcome. The results were very similar to the results of the mediation model predicting work engagement. SMC was significantly related to ASCS-W, and ASCS-W was significantly related to PWB. Thus, Hypothesis 3a was also confirmed. The indirect effect of SMC via ASCS-W on PWB was also significant which is indicated by the result of a Sobel-Test and the bootstrap confidence interval.

**Fig. 4:**
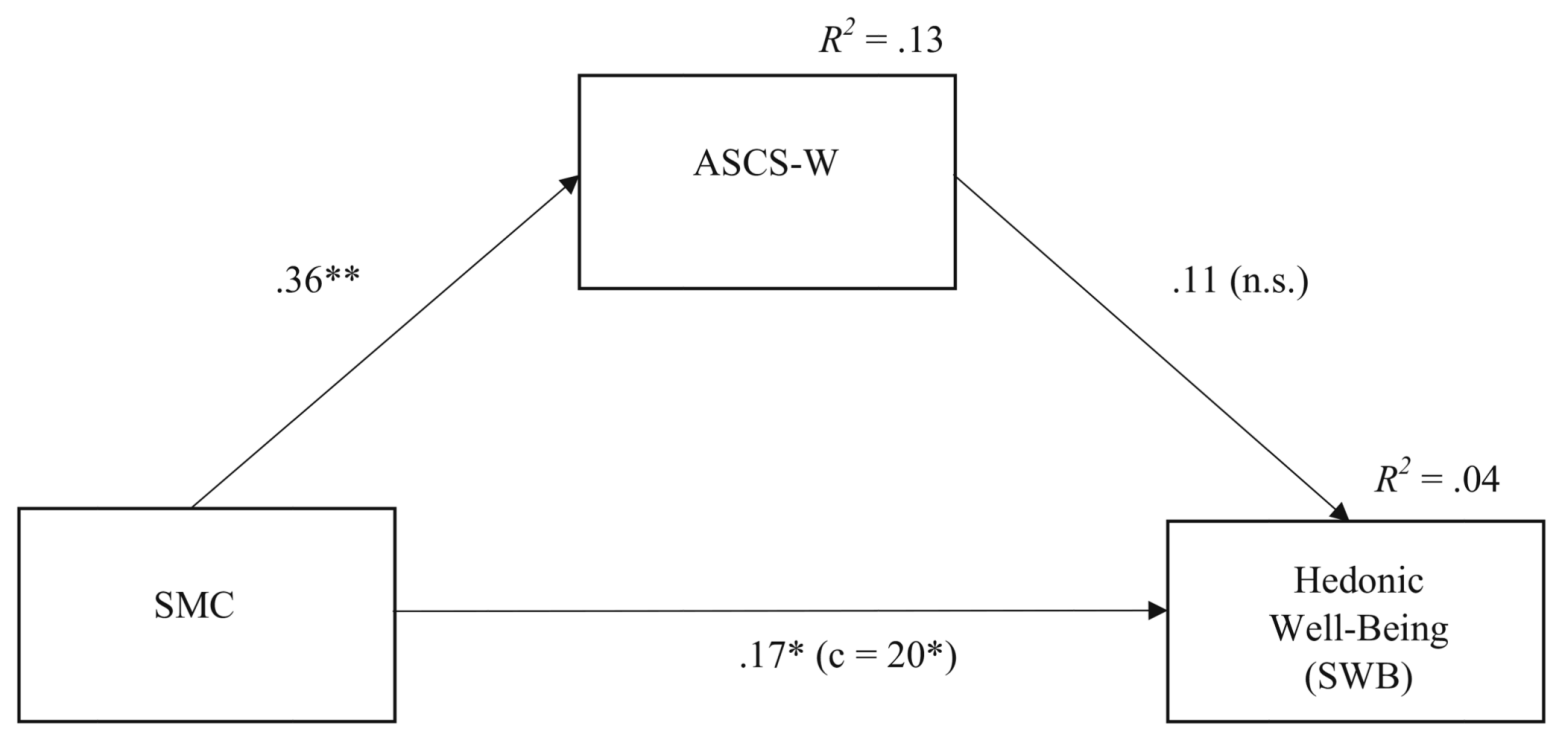
Results for the mediation model predicting SWB (T1). *Note. N =* 165; *p < .05, ** *p* < .01, n.s.: non-significant (one-tailed); standardized regression coefficients; SMC: Socio-moral climate; ACSW: Applicability of signature character strengths at work*; R^2^* = determination coefficient (explained variance); c: total effect without controlling for indirect effect

The third cross-sectional mediation model comprised SWB as outcome. Results are depicted in Table 4 and Fig. 4. We found no significant relationship between ASCS-W and SWB based on this multivariate analysis controlling for SMC. However, the bivariate correlation between ASCS-W and SWB was significant (see Table 1). For testing Hypothesis 3c stating that the relationship between ASCS-W and PWB is stronger compared to the relationship with SWB we computed a difference test for the two bivariate correlations. The result (*z* = 3.34, *p* < .001) confirmed our Hypothesis 3c.

In line with the result of a non-significant relation between ASCS-W and SWB in the mediation model, the indirect effect of SMC on SWB was not significant, either. However, we found a weak but significant direct effect of SMC on SWB.

Based on the longitudinal sub-sample, we tested Hypothesis 4, which stated an effect of SMC on ASCS-W over time. Before performing this analysis, we tested whether mean scores of SMC and ASCS-W at T1 significantly differed between physicians participating at T1 and T2 from mean scores of those physicians which had only participated at T1. Results of *t*-Tests showed no significant differences between these two groups (SMC: *t*(163) = −0.05, *p* = .96; ASCS-W: *t*(163) = 1.45, *p* = .15).

**Fig. 5:**
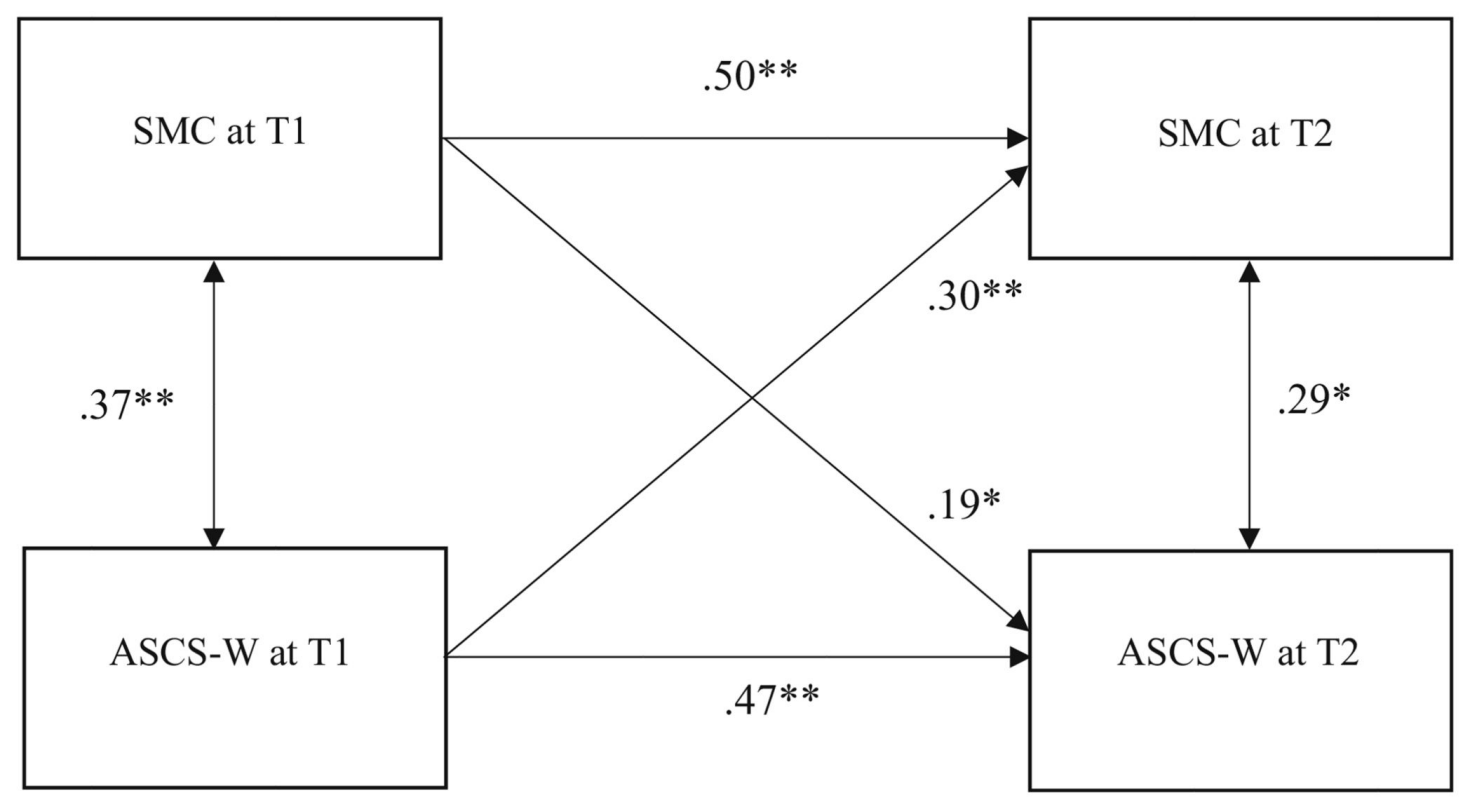
Results of the cross-lagged path-analysis for longitudinal relations between socio-moral climate and applicability of signature character strengths at work (T1/T2, time lag: 6 months). *Note. N* = 69. *p < .05, ** *p* < .01 (one-tailed), standardized regression coefficients; SMC: Socio-moral climate; ASCS-W: Applicability of signature character strengths at work

Figure 5 depicts the results of a cross-lagged path analysis with manifest variables. Because all variables were allowed to co-vary, and the model includes no other restrictions, fit indices for the model are not meaningful. The fit of a totally unrestricted model is always perfect by nature.

Supporting our hypothesis, the path-coefficient for the effect of SMC at T1 on ASCS-W at T2 was small but significant (*p* < .05). However, unexpectedly we also found a significant – and even stronger – cross-lagged effect of ASCS-W at T1 on SMC at T2.

## Discussion

This study is one of the first exploring a potential condition-related antecedent of the applicability of signature character strengths at work (see also Strecker et al. 2018 in this special issue). The importance of investigating condition-related factors in positive psychology has already been emphasized by Seligman and Csikszentmihalyi (2000), and with the narrower focus on the development and display of character- and other strengths by Peterson and Seligman (2004) as well as Stokols (2003). Moreover, this study replicated previous findings concerning the positive relations between the applicability of signature character strengths at work, work engagement and well-being (e.g., Lavy and Littman-Ovadia 2017; Linley et al. 2010; Harzer and Ruch 2013) in a sample of hospital physicans.

Based on cross-sectional data we demonstrated that the perceived socio-moral climate (SMC) of the medical department was positively related to the applicability of signature character strengths at work (ASCS-W), which in turn was positively related to physicians’ work engagement and the eudaimonic component of well-being (PWB). Thus, perceived SMC affected work engagement and PWB indirectly via increased ASCS-W. This result supported the idea of SMC as an antecedent of ASCS-W and additionally suggested a psychological mechanism of how SMC affects work engagement and PWB.

However, in the mediation model we found no significant effect from ASCS-W on the hedonic component of well-being (SWB). In addition, the bivariate correlation between ASCS-W and SWB (*r* = .17; *p* < .05; Table 1) was weaker compared to the correlation between ASCS-W and PWB (*r* = .35; *p* < .01; Table 1). A closer association of ASCS-W with PWB than with SWB is in line with the results of Hausler et al. (2017b), indicating that ASCS-W is more strongly linked to facets of well-being that are associated with experiences of self-actualization, fulfillment and meaningfulness than with the mere presence of positive emotions, the absence of negative emotions and general life satisfaction (SWB). It could be a promising route for research to investigate possible moderator variables for the link between ASCS-W and SWB. For example, Allan and Duffy (2014) demonstrated in a study with undergraduate students that the association between signature strength use and academic satisfaction was weaker among students experiencing high levels of *calling*.

Concerning the hypothesized antecedent function of SMC for ASCS-W, the results of the cross-lagged-panel path analysis showed that the relationship between both constructs might be more complex than initially proposed. Indeed, we found that perceived SMC at T1 was not only linked to ASCS-W at T1 but also predicted ASCS-W at T2 6 months later (controlled for ASCS-W at T1). However, we also detected the reversed effect that ASCS-W at T1 predicted SMC at T2 (controlled for SMC at T1). This effect was even stronger than the effect of SMC on ASCS-W over time. There are at least two conclusions: Firstly, the results can be interpreted as a hint of the existence of a positive gain spiral: A highly developed socio-moral climate may lead to a better applicability of signature character strengths of employees, which in turn may affect the socio-moral climate at work positively and so on. However, to test such a gain spiral directly, more than two measurements are needed. Secondly, the results shed light on the evolution of the socio-moral climate at work. The climate of a work unit (e.g. team, department, organization) can be seen as the shared perceptions of the sum of all behaviors of all agents within this social unit. Thus, employees displaying their own positive character strengths at work contribute to the climate of the work unit positively by affecting it through their own behavior. Or in other words: Employees’ behaviors are not only influenced by the socio-moral climate of their work unit but also actively create the socio-moral climate.

This study has some limitations to be considered. SMC of the medical department was measured only as perceived SMC on the individual level and only individual level effects were analyzed. Future research should apply multilevel designs and multilevel analysis (Bryk and Raudenbush 1992; Kozlowski and Klein 2000) focusing on the effects of the aggregated (shared) perceptions of SMC within each unit (Glick 1985) and their cross-level effects on individual ASCS-W and well-being. Unfortunately, our sample did not include an adequate number of different medical departments to apply such a multilevel approach (Maas and Hox 2005). A further limitation is the operationalization of signature character strengths exclusively by their ipsative ranks (top five). Although this operationalization of signature character strengths is very common in the empirical literature (e.g., Allan and Duffy 2014; Harzer and Ruch 2012, 2013; Linley et al. 2007, 2010) signature character strengths originally are defined by more than the mere rank (Peterson and Seligman 2004): e.g., sense of ownership and authenticity (‘this is the real me’), feelings of excitement, invigoration and inevitability when displaying them. In future research, these additional criteria could be operationalized by an additional scale or by questions from an existing structured interview (VIA-SI, Peterson 2003).

Furthermore, the longitudinal study included only two measurements and the sample was small. Therefore, it was not possible to test the whole mediation model longitudinally and to analyze possible reversed effects (gain spiral, see above) in more detail. Future research should apply more rigorous longitudinal designs with more repeated measurements (at least three). Moreover, intervention studies are also needed to establish causality.

A further limitation of the study was that all data were collected as self-reports increasing the risk of a common method bias resulting in inflated correlations (Podsakoff et al. 2003).

Finally, we collected our data in a very specific occupational field: hospital physicians. Thus, a generalization of results to other occupational contexts is questionable.

Against the background of these limitations, further research should primarily focus on applying multilevel longitudinal designs with at least three measurement points and multiple occupational contexts. Future research should not only analyze the relation between SMC and the applicability of individual signature character strengths but also the applicability of *specific* character strengths (Littman-Ovadia et al. 2017). It should also expand the focus to other personal strengths besides character strengths (Huber et al. 2017). Furthermore, it is important not only to investigate SMC and the applicability of existing character strengths but also to explore whether SMC contributes to the development of character strengths via the fostering of moral orientations and behaviors (Weber et al. 2009).

Our study was the first one to investigate SMC in a hospital setting. Thus, it also has practical implications, which refer to possible interventions in hospitals at the organizational and the individual level. On the organizational level the results indicate that improving SMC in medical departments is a promising route to enhance the use of character strengths and to improve work engagement and well-being of hospital physicians. Previous research on SMC identified two important antecedents of SMC at work: Participative organizational structures (Weber et al. 2008) and a servant leadership style (Pircher Verdorfer et al. 2015), which can be targets of organizational change processes in the hospital setting. Of course, both change processes are promising but also particularly challenging endeavors in this special field, because hospital physicians’ work -at least in German speaking countries- is traditionally characterized by comparatively strong hierarchy and directive leadership styles which may affect SMC negatively. This might be the reason that the mean of SMC in our hospital sample (*M* = 2.73, *SD* = .78) was notably lower compared to SMC-levels in samples from other occupational fields in German speaking countries (Germany, Austria, Switzerland; Pircher Verdorfer et al. 2015; Seyr and Vollmer 2014; Weber et al. 2008) and the USA (Pircher Verdorfer et al. 2015).

At the individual level the results of our study are indicative of the development and the application of person-centered character strength interventions (Quinlan et al. 2012). Hospital physicians could be instructed how to use their signature character strengths at work proactively. Considering our results concerning the cross-lagged effects, this should not only have positive consequences for hospital physicians’ work engagement and well-being directly but also lead to a further improvement of SMC.

## Figures and Tables

**Table 1:** Means, standard deviations, intercorrelations.

		*M*	*SD*	1	2	3	4	5	6	7	8	9
1	Sex (1 = female; 2 = male)	–	–									
2	Age (T1)	33.44	6.73	.00								
3	SMC (T1)	2.73	0.78	.01	–.07	(.93)						
4	SMC (T2)*	2.58	0.75	.03	–.15	.60**	(.91)					
5	ASCS-W (T1)	3.89	0.53	–.03	.09	.37**	.48**	(.80)				
6	ASCS-W (T2)*	3.76	0.57	.05	–.03	.33**	.52**	.55**	(.79)			
7	Work Engagement (T1)	3.65	1.12	–.03	–.05	.27**	.29*	.43**	.37**	(.94)		
8	PWB (eudaimonic) (T1)	3.85	0.40	–.09	–.01	.23**	.35**	.34**	.45**	.55**	(.92)	
9	SWB (hedonic) (T1)	3.92	0.73	–.06	.02	.20*	.28*	.17*	.28*	.52**	.77**	(.96)

Pearson-correlations T1/T1*: N* = 165; Pearson-correlations T1/T2 and T2/T2: *N* = 69; *p < .05, ** *p* < .01 (two-tailed) *M* Mean, *SD* standard deviation, *matrix diagonal* Cronbach’s Alpha (scale reliability), *SMC* Socio-moral climate, *ASCS-W* Application of signature character strengths at work, *PWB* Psychological well-being (eudaimonic), *SWB* Subjective well-being (hedonic)

**Table 2:** Mediation model predicting work engagement (T1)

Outcome: Work Engagement	β	*p*	95% CI
SMC ➔ Work Engagement (total effect)	.27	<.001	[.12, .42]
SMC ➔ ASCS-W	.37	<.001	[.22, .51]
ASCS-W ➔ Work Engagement^b^	.38	<.001	[.23, .53]
SMC ➔ Work Engagement (direct effect)	.13	.04	[.001, .28]
SMC ➔ ASCS-W ➔ Work Engagement (indirect effect)	.14	<.001^a^	[.07, .23]

*N* = 165 *SMC* Socio-moral climate, *ASCS-W* Applicability of signature character strengths at work, *β* Standardized regression coefficient, *p* probability level (one-tailed), *95% CI* bootstrap confidence interval (95%)

a Sobel Test

b controlled for SMC

**Table 3:** Mediation model predicting PWB (T1).

Outcome: PWB (eudaimonic)	β	*p*	95% CI
SMC ➔ PWB (total effect)	.23	.002	[.07, .38]
SMC ➔ ASCS-W	.36	<.001	[.22, .51]
ASCS-W ➔ PWB^b^	.29	<.001	[.13, .45]
SMC ➔ PWB (direct effect)	.12	.07	[–.04, .29]
SMC ➔ ASCS-W ➔ PWB (indirect effect)	.11	.004^a^	[.04, .21]

*N* = 165 *PWB* Psychological well-being (eudaimonic), *SMC* Socio-moral climate, *ASCS-W* Applicability of signature character strengths at work, *β* Standardized regression coefficient, *p* probability level (one-tailed), *95% CI* bootstrap confidence interval (95%)

a Sobel Test

b controlled for SMC

**Table 4:** Mediation model predicting SWB (T1).

Outcome: SWB (hedonic)	β	*p*	95% CI
SMC ➔ SWB (total effect)	.20	.01	[.05, .36]
SMC ➔ ASCS-W	.36	<.001	[.41, .62]
ASCS-W ➔ SWB^b^	.11	.10	[–.06, .27]
SMC ➔ SWB (direct effect)	.17	.03	[.001, .33]
SMC ➔ ASCS-W ➔ SWB (indirect effect)	.04	.12^a^	[–.03, .11]

*N* = 165 *SWB* Subjective well-being (hedonic), *SMC* Socio-moral climate, *ASCS-W* Applicability of signature character strengths at work, β Standardized regression coefficient, *p* probability level (one-tailed), *95% CI* bootstrap confidence interval (95%)

a Sobel Test

b controlled for SMC
